# Stochasticity in economic losses increases the value of reputation in indirect reciprocity

**DOI:** 10.1038/srep18182

**Published:** 2015-12-14

**Authors:** Miguel dos Santos, Sarah Placì, Claus Wedekind

**Affiliations:** 1 Department of Ecology and Evolution, Biophore, University of Lausanne, 1015 Lausanne, Switzerland

## Abstract

Recent theory predicts harsh and stochastic conditions to generally promote the evolution of cooperation. Here, we test experimentally whether stochasticity in economic losses also affects the value of reputation in indirect reciprocity, a type of cooperation that is very typical for humans. We used a repeated helping game with observers. One subject (the “Unlucky”) lost some money, another one (the “Passer-by”) could reduce this loss by accepting a cost to herself, thereby building up a reputation that could be used by others in later interactions. The losses were either stable or stochastic, but the average loss over time and the average efficiency gains of helping were kept constant in both treatments. We found that players with a reputation of being generous were generally more likely to receive help by others, such that investing into a good reputation generated long-term benefits that compensated for the immediate costs of helping. Helping frequencies were similar in both treatments, but players with a reputation to be selfish lost more resources under stochastic conditions. Hence, returns on investment were steeper when losses varied than when they did not. We conclude that this type of stochasticity increases the value of reputation in indirect reciprocity.

Understanding costly cooperation, i.e. why and under what conditions would someone pay a cost so that someone else receives a benefit, is still a major problem in evolutionary biology. Among the key factors that can lead to cooperation is discrimination between cooperative and uncooperative partners^1^,^2^. Such discrimination is based on the behavior of others as experienced in direct interactions (e.g. in direct reciprocity^3^), or on their behavior towards third parties that is then translated into some kind of reputation as, for example, in indirect reciprocity^4^,^5^. In indirect reciprocity, helping someone or refusing to do so, and even punishing someone for not helping, builds up a reputation that is likely to influence others within a social group^6^,^7^,^8^,^9^. Humans have shown cooperative behavior in various kinds of experimental indirect reciprocity games, i.e. they can base their helping behavior on the reputation of others^10^,^11^,^12^,^13^,^14^,^15^,^16^,^17^,^18^. In addition, cooperative behaviors have been shown to be influenced by a number of implicit and explicit features that induce players to care about their reputation within their social group^19^, such as eye-like spots in the background^20^ or salience of group identity^21^. However, the computational algorithms that humans use in such games are not understood, and it is unclear how humans integrate the various characteristics that define their environment.

A particularly important feature of many environments is that they are not stable. Climate, resource availability, and the presence of predators or diseases are among the most common sources of environmental stochasticity that affect survival and reproduction. Adversity and stochasticity of the environment (defined as the mean and variance in environmental quality, respectively) have been shown to increase cooperation in plants and animals, for example, with rising elevation and physical stress in mountain areas^22^, with living in semiarid savanna habitats and high temporal variability in rainfall^23^, with increasing predation risks, especially in environments that provide little protection^24^,^25^, or with growing uncertainty about a nearby predator’s intentions^26^,^27^. These results are intriguing from an evolutionary point of view, because the costs of unreciprocated cooperation, and hence the risks linked to a generous act, may increase with the level of environmental stress^28^. Theoretical analyses of the problem concluded that increased environmental adversity and uncertainty can indeed lead to higher levels of cooperation in groups of selfish individuals^28^,^29^. Cooperation seems to be one way to counterbalance unforeseen fitness decrease due to environmental conditions^29^.

In humans, both environmental adversity and stochasticity seem to increase within-group solidarity and resource sharing^30^,^31^. However, it is still unclear whether and how indirect reciprocity is affected by the different forms of environmental stochasticity in social interactions (e.g. environment quality, payoff structure or frequency of interactions). Here we focus on stochasticity in loss of resources.

## Methods

### Ethics statement

All participants were recruited from a pool of volunteers of the Department of Economics of the University of Lausanne using ORSEE^32^. Participants were first year students from all fields of the University of Lausanne and the Swiss Federal Institute of Technology in Lausanne. The experiments were approved by the ethics committee of the University of Lausanne on the use of human subjects in research. Each participant signed an informed consent describing the nature of the experiment before entering the laboratory. Participants were told that their anonymity would be ensured throughout the game, as their decisions could not be linked with their real identity, neither by the other participants, nor by the experimenter. The experiments were carried out in accordance with the approved guidelines.

A total of 144 participants were distributed to 16 separate groups of 9. In order to play anonymously within groups, players were asked to choose a plug from an impenetrable tangle of cables, connect it to a box, and choose one of 9 isolated cubicles in juxtaposition from where they could all see the same screen that displayed the details of the game. To reveal a choice, players could secretly push one of two buttons inside the box. The buttons were connected via cables and a switchboard to a green and a red light, respectively^11^,^18^. These lights (i.e. decisions) were only revealed to the experimenter, who then entered the decisions in the computer in order to show them on the general display and to compute the players’ decision history (see Supplementary Material). Player IDs were distributed (and later gains paid out) in a procedure that ensured full anonymity, following the procedure dos Santos *et al.*^18^ used. The experimenter then read the game instructions (supplementary material) while they were also displayed on the main screen. Each player received an initial endowment of 35 Swiss francs (CHF) that was the starting capital for the game. They were told that they would, after the game had finished, be paid out whatever was left of this or gained to it, in addition to a guaranteed show-up payment of 10 CHF. No information was given about the total number of interactions that would be played.

Eight groups each played a pair-wise indirect reciprocity game in a “*Stable*” or “*Stochastic*” treatment. At each interaction, one player was put in the “*Unlucky*” role and lost 4 CHF (*Stable*) or either 3 or 5 CHF (*Stochastic)*. Another player, put in the “*Passer-by*” role, had to decide whether or not to reduce this loss to 1 CHF (i.e. to help the *Unlucky*) by accepting a cost of 1 CHF to herself^33^. Then a new pair of players was put in these two roles. Players were told that the same pair would never play in the reversed role, i.e. direct reciprocity was not possible (as a consequence, each player could only be in the *Passer-by* role for 4 of the group members, and in the *Unlucky* role for the other 4 group members). At each interaction, the *Unlucky’s* history of giving or not giving in the *Passer-by* role (i.e. her reputation) was graphically displayed with a pile of circles of 2 different sizes and 2 different colors (supplementary material): giving something (not giving) was indicated with a blue (yellow) circle, and giving something to an *Unlucky* who lost 3 (5) was indicated by a small (large) circle. Giving or not giving to an *Unlucky* who lost 4 was indicated by a medium sized circle. On the display, the history of giving or not giving could potentially comprise 25% more circles than the total number of rounds that were actually played in order to avoid that players could infer the total number of rounds, i.e. to avoid potential end-game effects. We decided to display the full history of the *Unlucky*’s helping behavior in the role of the *Passer-by* to avoid introducing assumptions about how humans process information about previous choices of others.

Each player played 24 times in each role. Hence, each player was paired 6 times with each recipient or donor. In the *Stochastic* treatment, each player was 12 times the *Unlucky* with a 3 CHF loss and 12 times the *Unlucky* with a 5 CHF loss. Also, each player played 12 times as the *Passer-by* with an *Unlucky* losing 3 CHF and 12 times with an *Unlucky* losing 5 CHF, i.e. the experimental design was fully balanced with respect to the kind of losses experienced in both roles. The order of the type of losses was randomized, and participants were not made aware of the balanced nature of the design.

The participants’ payoff during the game was not displayed in order to avoid potential envy effects. In total, each of the 9 players of a group had 48 interactions, i.e. the total number of pairwise interactions was 216 (i.e. 48*9*0.5). In order to avoid negative balances, all players (including the observers) received 0.25 CHF after each interaction. Therefore, at the end of the game, each player had received a total of 54 CHF (i.e. 216*0.25) in addition to their payoff during the game and the show-up payment. This amount was added gradually during the game to avoid potential effects of high initial endowments on the players’ decisions.

The statistical analyses were carried out with R 2.10.1^34^. We used the ‘lme4’ package^35^ for linear (LMM) and logistic mixed-effect model (GLMM) analyses. Whenever LMM were used, the group identity was included as a random effect. To control for the robustness of the results using LMMs, we re-fitted these models as described in Campell and Walters^36^, using linear regressions with robust standard errors (with group identity as cluster) and the ‘sandwich’ package^37^. P-values obtained with this method are denoted by *p*_rob_. The *Passers-by*’s probability of giving was analyzed using GLMM with group and individual as random effects.

In the *Stable* treatment, the *Unlucky*’s reputation at a given interaction was computed as her cooperation frequency minus the group mean cooperation frequency until that interaction in order to correct for group and time effects. Qualitatively similar results were obtained using the absolute cooperation frequency, however higher AICs were found using the latter, suggesting that the models’ quality of fit was lower (Supplementary Table 2).

In the *Stochastic* treatment, the *Unlucky*’s reputation was computed analogously (i.e. based on the frequency of blue circles). We did not split this variable into one reputation towards *Unluckies* suffering a small loss and one reputation towards *Unluckies* suffering a large loss as these two variables were correlated (corrected for group and round effects: Spearman’s rank correlation coefficient rho = 0.36, *p* < 0.0001). In order to further examine their combined effect on the *Passer-by*’s decision, we first computed the *Unlucky*’s reputation as her cooperation frequency towards *Unluckies* suffering a large loss, and added to the GLMM a variable ‘Discrimination’ representing the difference in cooperation frequency between when *Unluckies* were suffering a large loss and when they were suffering a small loss (a positive difference would mean that the focal player helped more often *Unluckies* suffering a small loss than those suffering a large loss). The variable ‘Discrimination’ had only an additive effect (GLMM: discrimination, 2.29 ± 0.39 SE, *p* < 0.001), the interaction with reputation towards *Unluckies* suffering a large loss was not significant (GLMM: −0.68 ± 0.71 SE, *p* = 0.33). We therefore favored the simpler model with the overall cooperation frequency.

## Results

We found high proportions of helping in both treatment conditions (*Stable*: mean = 76.3%, range = 55–95%; *Stochastic*: mean = 70.1%, range = 45–88%) and no significant treatment effects on mean group cooperativeness (t-test on group means: t_14_ = 1.0, p = 0.33) or on the players’ final earnings (LMM: t = −0.68, p = 0.50, *p*_rob_ = 0.48). In the *Stochastic* treatment, the frequency of helping was higher if the *Unlucky* lost 5 CHF (635/864 donations; 73.5%) than if she lost 3 CHF (576/864 donations; 66.7%; paired t-test on group means, t_7_ = −3.67, p = 0.008; see Table 1 for an individual-based test). The overall efficiency gains from helping a needy partner (by reducing her loss) did not differ between treatments (t-test on group means, t_14_ = 0.68, p = 0.51).

The *Unlucky’s* reputation strongly influenced the *Passer-by*’s decisions in both, the *Stable* and the *Stochastic* treatments (Table 1a,b). A large loss in the *Stochastic* treatment increased the *Passer-by*’s probability of helping (Table 1b), but did not significantly affect the use of reputation (see the non-significant interaction between reputation and amount of loss in Table 1b). Whether the *Passer-by* was helped in the previous interaction did not seem to influence her decision in the *Stable* treatment (Supplementary Table 1a). In the *Stochastic* treatment however, this previous interaction may have affected the use of reputation, as *Passer-bys* who had not received were less likely to give, particularly to more generous *Unluckies* (Supplementary Table 1b; Supplementary Figure 1). The type of loss (i.e. large or small) suffered by the *Passer-bys* in their previous interaction seemed to have no effect here (Supplementary Table 1b).

**Figure 1:**
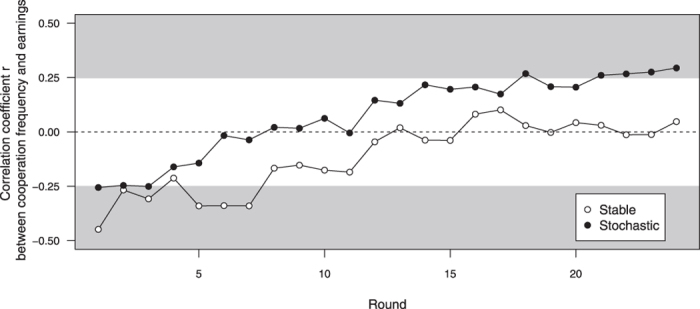
Pearson’s correlation coefficients r between cooperation frequency and earnings over time under *Stable* (open symbols) and *Stochastic* conditions (filled symbols). Correlation coefficients in the shaded area are significantly different from zero at *p* < 0.05, two-tailed.

**Figure 2:**
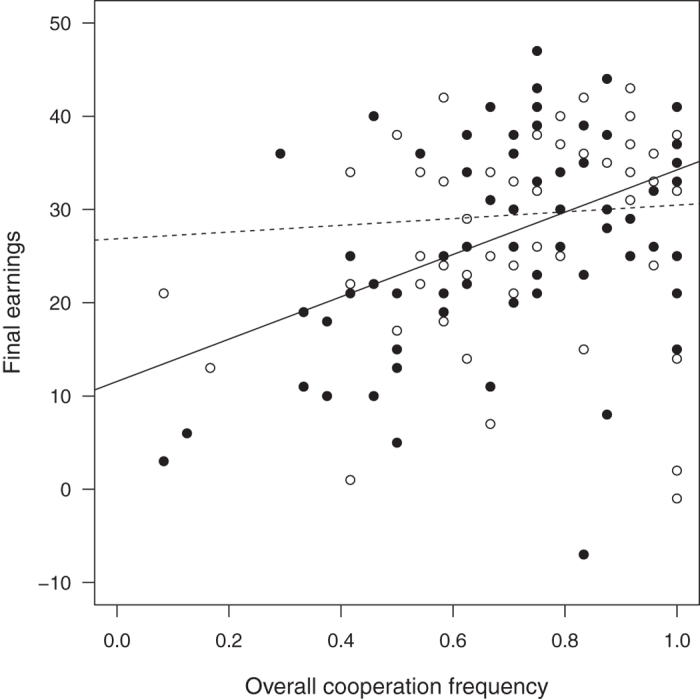
Regressions of cooperativeness on final earnings (Swiss francs) in the *Stable* treatment (open symbols, dashed line) and the *Stochastic* treatment (filled symbols, solid line). See text for statistics.

Figure 1 shows the relationship between the players’ generosity and their earnings over time. As expected, the correlation between generosity and earnings was negative at the start of a game (reflecting the immediate costs of generosity). Over time, the *Passer-bys’* tendency to reward a reputation of being generous increasingly compensated for the costs of generosity in both treatments (Fig. 1). However, the return on investment into reputation was steeper in the *Stochastic* than in the *Stable* treatment, as shown by the positive relationship between final earnings and final helping frequency at the end of the 24 rounds in the *Stochastic* treatment (LMM on final helping frequency corrected for group effects: slope = 12.1 ± 5.96 SE, *p* = 0.044, p_rob_ = 0.033) but not in the *Stable* treatment (slope = −5.83 ± 7.33 SE, *p* = 0.43, p_rob_ = 0.31; slope difference between *Stable* and *Stochastic* = −17.94 ± 9.45, *p* = 0.06, p_rob_ = 0.028. Not correcting for possible group effects led to qualitatively similar results (Fig. 2).

The underlying factor for the difference in return on investment into reputation between our treatments is likely due to the fact that more selfish players within groups seem to have received help less often under *Stochasticity* than under *Stable* conditions, as shown by explorative analyses based on a *post-hoc* categorization of players into ‘selfish’, ‘medium’, and ‘generous’ reputation (Supplementary Figures S1). As a consequence, it seems that players categorized as selfish lost higher amounts when in the *Unlucky* role under *Stochasticity* than under *Stable* conditions (Supplementary Figures S2).

## Discussion

We tested whether adding stochasticity on future economic losses incurred by individuals playing an indirect reciprocity game affected cooperation and/or the use of information on group members’ past behaviors. We found similar cooperation levels between stable environments, where losses endured by individuals were perfectly predictable, and stochastic environments, where losses varied (while overall losses were always kept constant by the experimental set up). Also, donations turned out to be more frequent to those who had been previously generous to others under both treatments, confirming previous observations under experimental and field conditions^10^,^11^,^12^,^13^,^14^,^15^,^16^,^17^. However, when deciding to help needy group members, people were differently influenced by their partners’ past behaviors with others. Under stable conditions, the tendency to reward generosity only allowed generous players to compensate for the cost of helping others, as there was no correlation between reputation (i.e. information on past behaviors with others) and final earnings. In other words, investing into a good reputation did not generate enough benefits for generous group members to outperform more selfish ones. Under stochastic conditions, however, selfish players within groups were helped relatively less often and therefore finished with lower payoffs than more generous group members. As a consequence, the steepness of the return on investment critically depended on the environment: investing into a good reputation paid back earlier under stochastic than under stable conditions.

Our findings suggest that people are less forgiving with selfish members of their group when harmful events in the environment are unpredictable. One explanation could be that people merely expected higher levels of cooperation from others, and hence behaved more severely with selfish group members. However, our participants were not only more severe with uncooperative players, but also, not being helped affected their decisions with future partners (a concept known as ‘generalized reciprocity’ or ‘paying it forward’^38^,^39^). An alternative explanation could be that interacting in an unpredictable environment elicited (more) stress in our participants than under stable conditions. In fact, it is well known in the psychological literature that unpredictability about future aversive events can be a major factor of stress for humans^40^, which could in turn affect decision making^41^,^42^. It has also been shown that people prefer predictable over unpredictable unpleasant stimuli, and that both conditions induce different types of neurobiological responses^43^,^44^. Anthropological studies have shown that solidarity between group members is higher in unpredictable environments^30^. It is possible that cognitive mechanisms were selected in early humans to adjust their cooperative expectations in stressful conditions^29^. In our experiments, the more stressful nature of a stochastic environment might have led players to perceive differently both uncooperative players and not receiving help when in need, which eventually led to different behaviors. An interesting line of research would be to test whether other factors of stress, e.g. time pressure^45^, also affect the use of reputation in a similar way. It would also be interesting to see if relative cooperation frequency (i.e. score within a group) is indeed more important in indirect reciprocity than the absolute cooperation frequency, as suggested by the higher AICs we found for the models using relative reputation scores. If so, this would further support the hypothesis that humans generally assess their partners’ relative quality and generosity in biological markets^46^,^47^.

At the ultimate level, our results suggest that stochasticity could be a catalyzer for the evolution of cooperation through indirect reciprocity. In fact, the evolution of cooperation through a reputation system requires that the costs of investment into a good reputation (i.e. the immediate costs of being generous) have to be, on average, well compensated by the effects the reputation has on the behavior of future social partners^5^, either within the indirect reciprocity game itself or when transferred to other contexts such as, for example, Prisoner’s Dilemma-type direct reciprocity games^11^,^48^. Hence, if individuals have already in place a hardwired psychological mechanism that makes them behave differently under stressful conditions (and which evolved for other reasons, e.g. coping with the environment), this could also affect their social interactions. Future theoretical work should investigate whether being less forgiving with selfish group members under environmental stochasticity would actually be adaptive, and in turn lead to the evolution cooperation more effectively.

Our analysis concentrated on whether or not the displayed information was taken into account and disregarded any other possible reputation-updating rules. We cannot exclude that players took higher-order information in account like, for example, the reputation of their partner’s previous recipients^49^. However, a previous experiment with a similar set-up (i.e. all interactions were observed by everybody) specifically tested for such higher-order strategies and did not find them to play a significant role^50^. Considering the number of interactions to observe and remember in our experiment, we believe it would probably have been cognitively too demanding, and hence unlikely, to frequently use higher-order information.

There is increasing evidence that adding different kinds of randomness to systems can have dramatic effects on the evolution of social behavior in various other contexts^28^,^29^,^51^. Stochastic evolutionary game theory can, for example, explain the extraordinary high levels of fairness that humans often display in Ultimatum Games^52^,^53^, or the evolution of the kind of overconfidence that seems very widespread in humans^54^. Indeed, randomness and uncertainty play important roles in human psychology, and recent experiments demonstrated that increased uncertainty led to higher offers in the Ultimatum Game^52^ and higher levels of trust in a Trust Game^55^. The finding that stochasticity in losses also increases the value of reputation in indirect reciprocity seems to fit well into this overall pattern and suggests that randomness can be an important driver also for the evolution of reputation-based behavioral strategies.

## Additional Information

**How to cite this article**: dos Santos, M. *et al.* Stochasticity in economic losses increases the value of reputation in indirect reciprocity. *Sci. Rep.* **5**, 18182; doi: 10.1038/srep18182 (2015).

## Supplementary Material

Supplementary Information

## Figures and Tables

**Table 1:** Indirect reciprocity under *Stable* and *Stochastic* conditions. Logistic regression on the *Passer-by*’s probability of giving in (a) *Stable* and (b) *Stochastic* conditions in function of the *Unlucky*’s reputation (i.e. helping frequency, relative to group and current interaction in order to correct for group and time effects) and current loss.

	Parameter estimate (±SE)	p
(a) *Stable* treatment		
Intercept	1.56 (0.34)	<0.001
* Unlucky’s* reputation	2.76 (0.35)	<0.001
(b) *Stochastic treatment*		
Intercept*	1.06 (0.30)	<0.001
* Unlucky’s* reputation*	3.31 (0.39)	<0.001
Large loss	0.47 (0.13)	<0.001
Reputation *x* Large loss	0.28 (0.53)	0.59

*Unluckies suffered a small loss.
