# Supplementary material for: Stochasticity in economic losses increases the value of reputation in indirect reciprocity
Source: Sci Rep. 2015 Dec 14;5:18182. doi: 10.1038/srep18182 (PMC4677356; doi:10.1038/srep18182)
Supplement: Supplementary Information [file srep18182-s1.pdf]

Supplementary Material

Stochasticity in economic losses increases the value of reputation in indirect reciprocity

Miguel dos Santos[1,2], Sarah Placì[1], Claus Wedekind[1]*

[1]Department of Ecology and Evolution, Biophore, University of Lausanne, 1015 Lausanne, Switzerland.

[2]Present address: Department of Zoology, University of Oxford, The Tinbergen Building, South Parks Road, Oxford, OX1 3PS, UK

*Corresponding author: Tel. +44 1865281987; miguel.dossantos@zoo.ox.ac.uk

Content:

- Supplementary Table S1 and S2
- Supplementary Figures S1 and S2
- Game instructions (translated from french)

Supplementary Table S1. Indirect reciprocity under *Stable* and *Stochastic* conditions. Logistic regression on the *Passer-by*'s probability of giving in both *Stable* and *Stochastic* conditions in function of the *Unlucky*'s reputation (i.e. helping frequency or generosity, relative to group and current interaction in order to correct for group and time effects) and current loss, as well as whether the Passer-by (Self), received in the previous interaction, and suffered a small or large loss in that previous interaction.

	Parameter estimate (± SE)	p
(a) *Stable* treatment		
Intercept*	1.78 (0.34)	< 0.001
Unlucky's reputation	3.96 (0.97)	< 0.001
Self received in last interaction	0.10 (0.16)	0.53
Reputation *x* Self received in last interaction	0.50 (0.85)	0.55
(b) *Stochastic treatment*		
Intercept*	0.99 (0.34)	0.003
Unlucky's reputation	2.70 (0.76)	0.004
Large loss	0.51 (0.14)	< 0.001
Self received in last interaction	0.40 (0.21)	0.06
Self suffered a large loss in last interaction	-0.19 (0.23)	0.41
Reputation *x* Large loss	0.14 (0.59)	0.81
Reputation *x* Self received in last interaction	2.14 (0.71)	0.002
Self received in last interaction *x* Self suffered a large loss in last interaction	0.28 (0.28)	0.32

*reference level when *Passer-by* did not receive in the previous interaction, and (b) when *Passer-by* suffered a small loss in the previous interaction, and *Unluckies* suffered a small loss

Supplementary Table S2. Qualities of fit, measured with Akaike information criterion (**AIC**) of models presented in Supplementary Table 1 (with relative reputation) compared with similar models, but with absolute reputation.

Treatment	Reputation	AIC
Stable		
	Relative (Table S1a)	1517.5
	Absolute	1522.8
Stochastic		
	Relative (Table S1b)	1571.7
	Absolute	1576.4

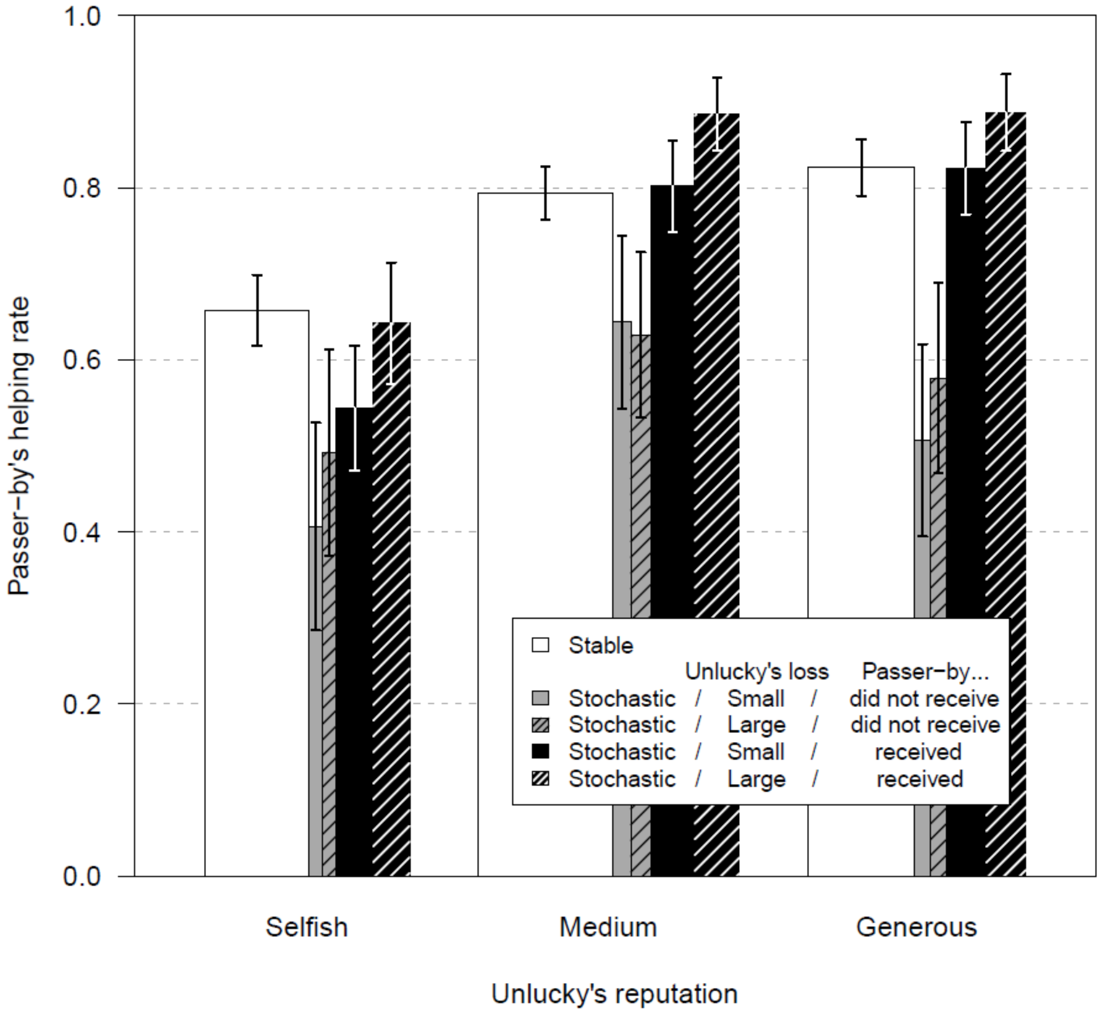

Figure S1. Rates of help received as a function of the *Unluckies*' reputation within their groups. Unluckies were ranked according to their generosity towards others as 'Generous' (ranks 1-3 in the group), 'Medium' (ranks 4-6), and 'Selfish' (ranks 7-9). The bars' width represents the number of constituent data points. A good reputation increased the likelihood of receiving help in both *Stable* and *Stochastic* conditions. Under *Stochastic* conditions, suffering a large cost and whether *Passer-bys* received help in the previous interaction increased the probability of receiving help. In addition, a large proportion of selfish players received less help than under *Stable* conditions. See Table 1 for statistics. Data shown represent means ± 95% confidence intervals of proportions.

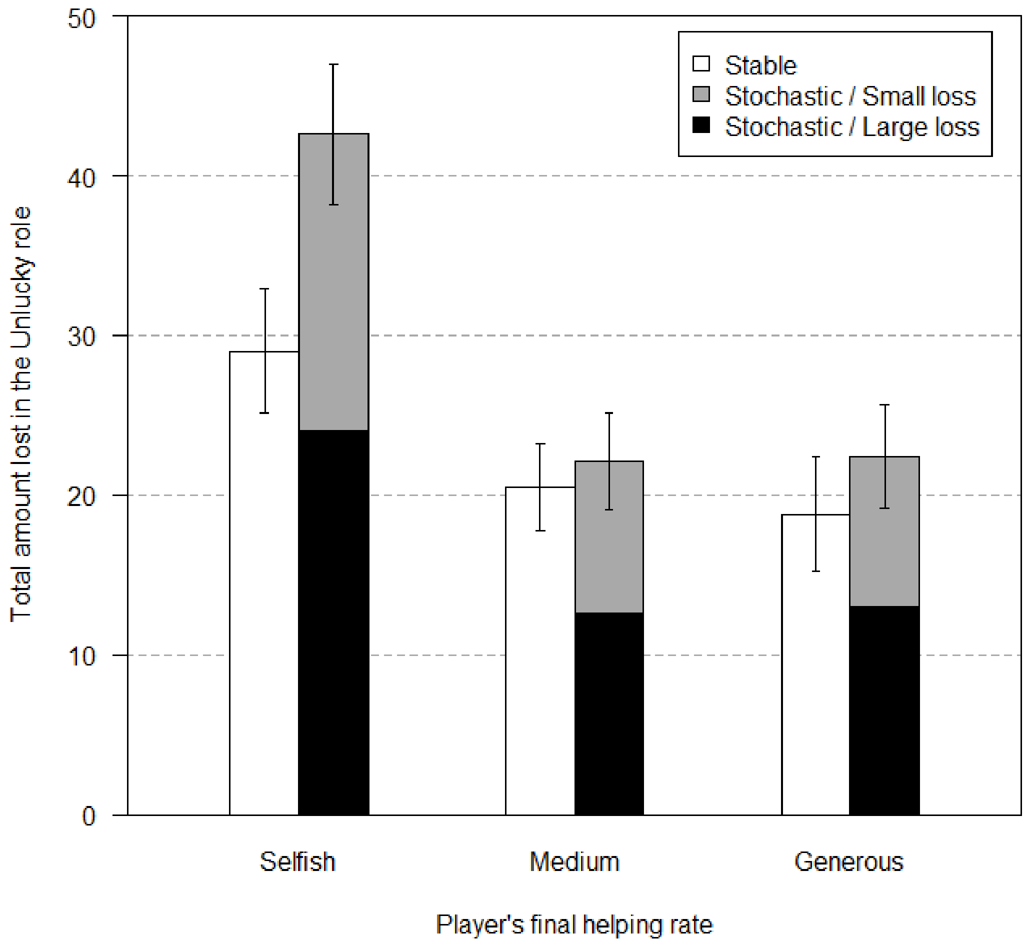

Figure S2. Total amount lost when in the *Unlucky* role under both *Stable* and *Stochastic* conditions as a function of final generosity. *Unluckies* are ranked in function of their generosity towards others as in Figure S1. Under *Stochastic* conditions, bars are separated by type of loss (i.e. small and large). Selfish players lost higher amounts (~19% more) under *Stochastic* conditions. Data shown represent means ± 95% confidence intervals. At the end of the game, selfish players received overall less help in the *Stochastic* than in the *Stable* treatment (Wilcoxon rank sum test on group means: w = 52.5, p = 0.035). Consequently, they lost significantly more money in the *Stochastic* than in the *Stable* treatment (Wilcoxon rank sum test on group means: w = 12, p = 0.038).

(Game instructions: *Stable treatment*)

Thanks for your participation to this study!

- You are going to play an economic game with the other participants
- Please follow the instructions carrefully
- If you have questions, please do not hesitate to ask us
- However, no communication will be allowed during the game

1

The game

- You will receive a show-up payment of 10 CHF, which is independent from your gains during the game
- You will receive, in addition, an initial endowment of 35 CHF which you can use during the game
- This is your money. You can keep it or use it in the game. You are going to have the opportunity to win or lose money **in function or your decisions and the decisions of the other participants**
- The money that you will earn will be transfered to you through a bank transfer

2

The game

- Your decisions will be made anonymously with the help of the carton box in front of you. The red button for «NO» and the white one for «YES»
- You will receive an identity number that nobody except yourself (not even the experimenter) will know
- Please never communicate this number, not even after the end of the game

3

The game

- The game will comprise many interactions
- In each interaction, two players are randomly selected, one the role of the *passer-by*, the other in the role of the *unlucky*.
- In each interaction, everybody will receive 0.25 CHF (selected players included)
- At the beginning of the interaction, the *unlucky* will lose 4 CHF
- The *passer-by* can decide to pay or not 1CHF for the *unlucky* to lose only 1CHF
- If the *passer-by* refuses, the *unlucky* will lose the initial loss
- You will never meet the same player in the reversed roles

4

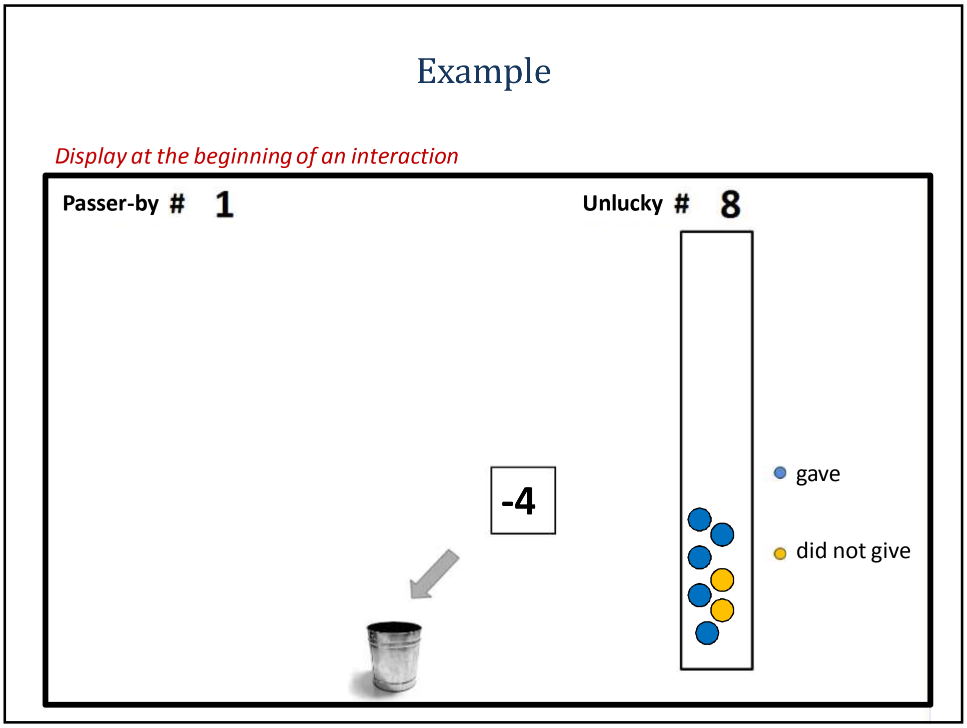
Example
Display at the beginning of an interaction
Passer-by # 1
Unlucky # 8
-4
gave
did not give

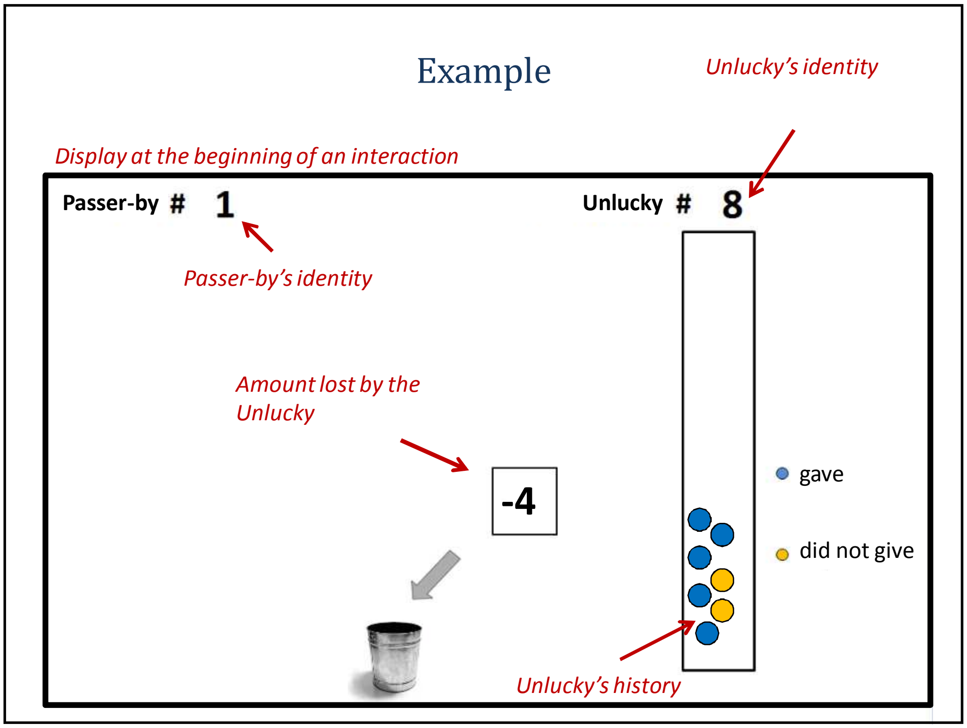
Example
Unlucky's identity
Display at the beginning of an interaction
Passer-by # 1
Unlucky # 8
Passer-by's identity
Amount lost by the Unlucky
-4
gave
did not give
Unlucky's history

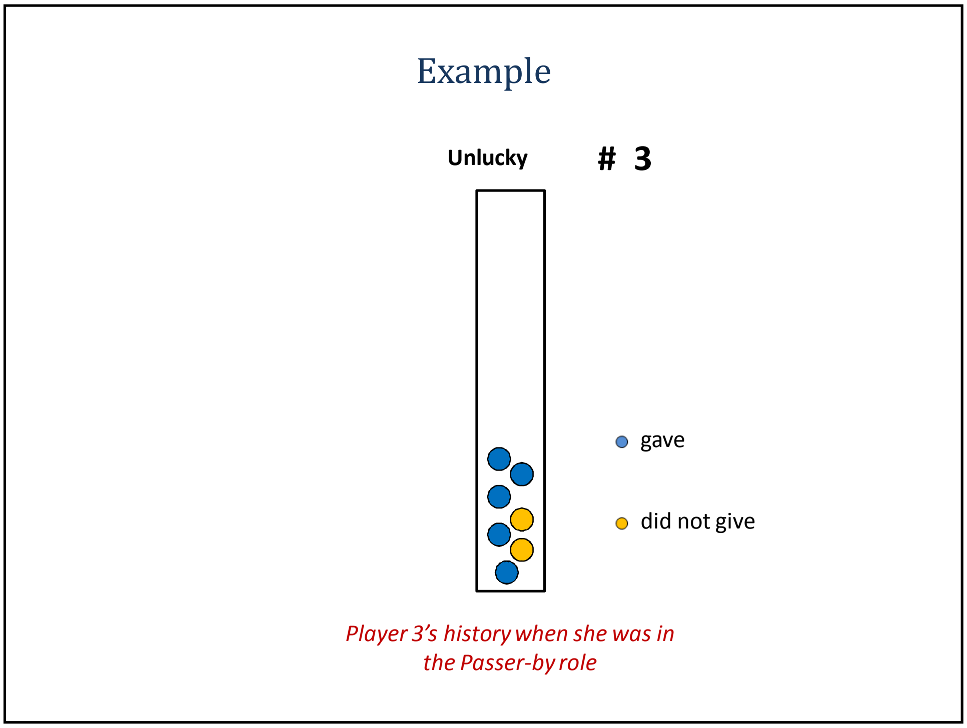
Example
Unlucky
3
gave
did not give
Player 3's history when she was in
the Passer-by role

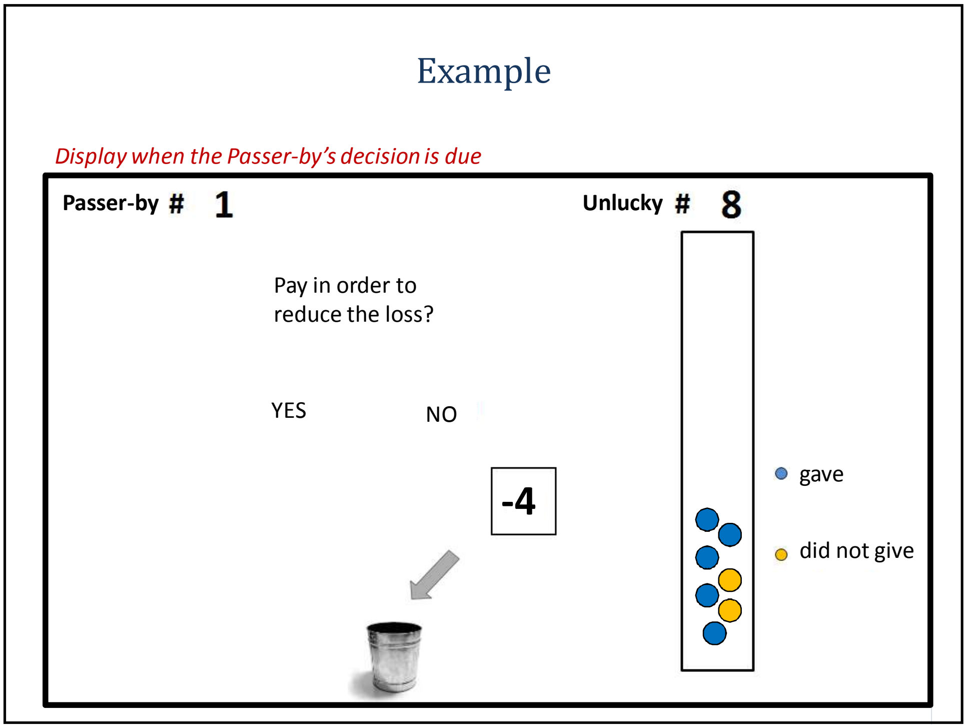
Example
Display when the Passer-by's decision is due
Passer-by # 1
Unlucky # 8
Pay in order to
reduce the loss?
YES
NO
-4
gave
did not give

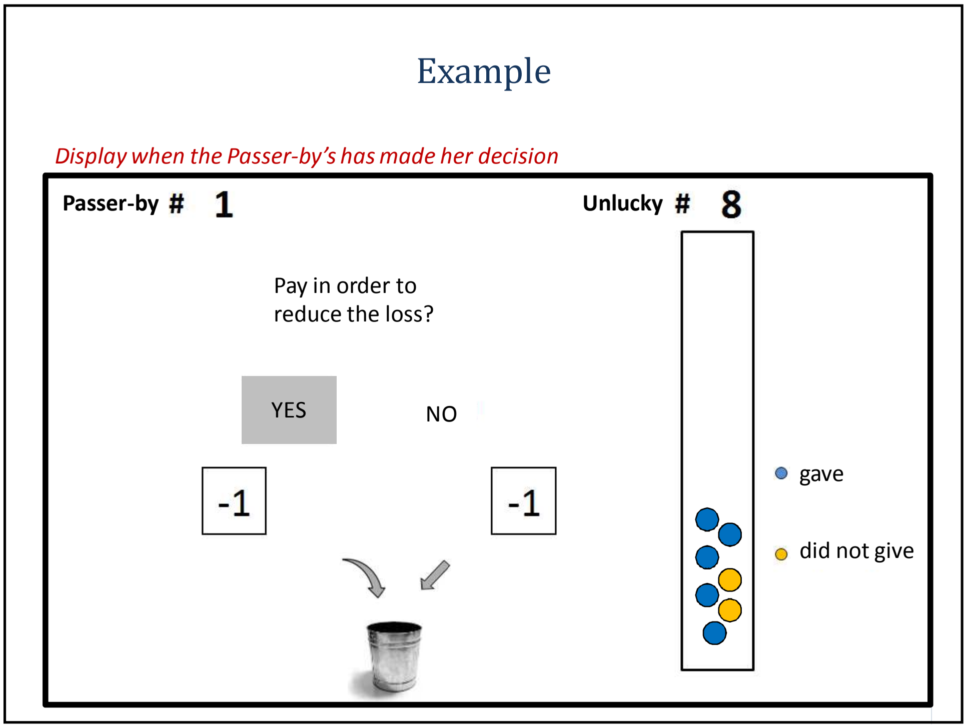
Example
Display when the Passer-by's has made her decision
Passer-by # 1
Unlucky # 8
Pay in order to
reduce the loss?
YES
NO
-1
-1
gave
did not give

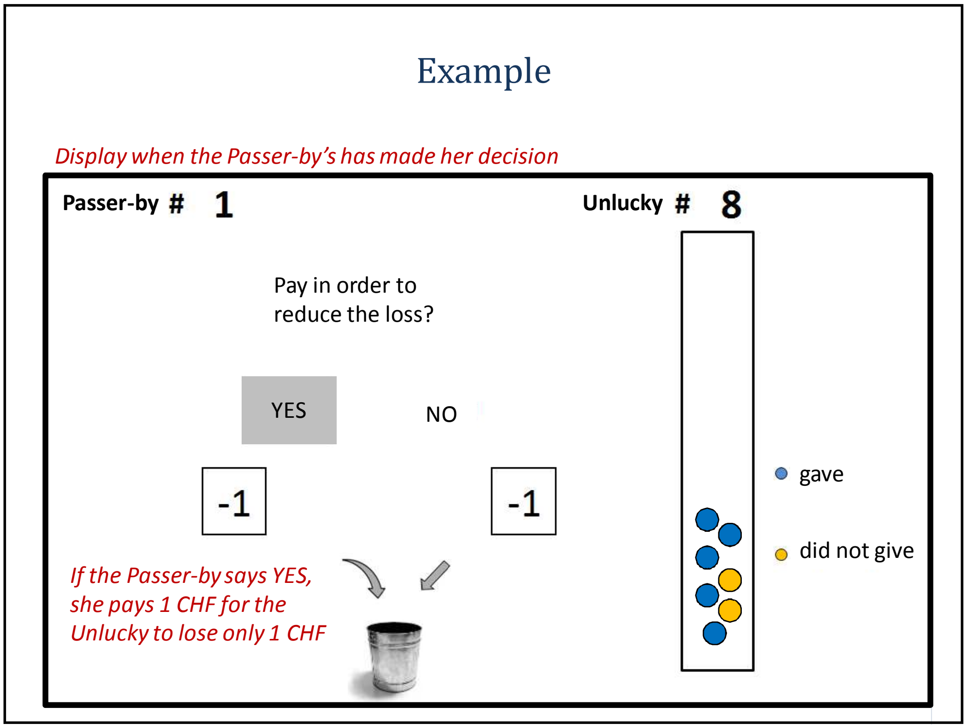
Example
Display when the Passer-by's has made her decision
Passer-by # 1
Unlucky # 8
Pay in order to
reduce the loss?
YES
NO
-1
-1
If the Passer-by says YES,
she pays 1 CHF for the
Unlucky to lose only 1 CHF
gave
did not give

Example

Display when the Passer-by's has made her decision

Passer-by # 1

Unlucky # 8

Pay in order to
reduce the loss?

YES

NO

0

-4

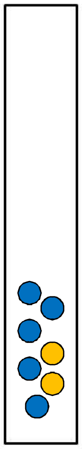

gave

did not give

If the Passer-by says NO, she pays nothing. The Unlucky loses the displayed amount

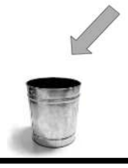

At the end of the game

- At the end of the game, you will be kindly asked to fill in the participation form and then place it in an envelope which will be sealed
- This envelope will only be opened by the accounting service of the Faculty of Biology in order to execute bank transfers
- You will learn about your final gains upon receipt on your bank account

12

Thanks for your attention !

Questions ?

13

(*Stochastic treatment*)

Thanks for your participation to this study!

- You are going to play an economic game with the other participants
- Please follow the instructions carrefully
- If you have questions, please do not hesitate to ask us
- However, no communication will be allowed during the game

14

The game

- You will receive a show-up payment of 10 CHF, which is independent from your gains during the game
- You will receive, in addition, an initial endowment of 35 CHF which you can use during the game
- This is your money. You can keep it or use it in the game. You are going to have the opportunity to win or lose money **in function or your decisions and the decisions of the other participants**
- The money that you will earn will be transfered to you through a bank transfer

15

The game

- Your decisions will be made anonymously with the help of the carton box in front of you. The red button for «NO» and the white one for «YES»
- You will receive an identity number that nobody except yourself (not even the experimenter) will know
- Please never communicate this number, not even after the end of the game

16

The game

- The game will comprise many interactions
- In each interaction, two players are randomly selected, one the role of the *passer-by*, the other in the role of the *unlucky*.
- In each interaction, everybody will receive 0.25 CHF (selected players included)
- At the beginning of the interaction, the *unlucky* will either lose 3 CHF or 5CHF (randomly determined)
- The *passer-by* can decide to pay or not 1CHF for the *unlucky* to lose only 1CHF
- If the *passer-by* refuses, the *unlucky* will lose the initial loss
- You will never meet the same player in the reversed roles

17

Example

Display at the beginning of an interaction

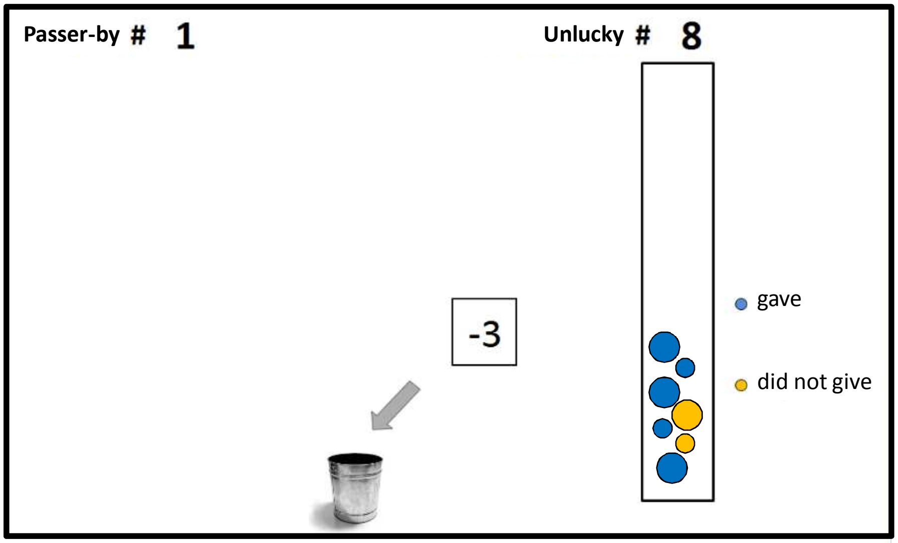

Example

Unlucky's identity

Display at the beginning of an interaction

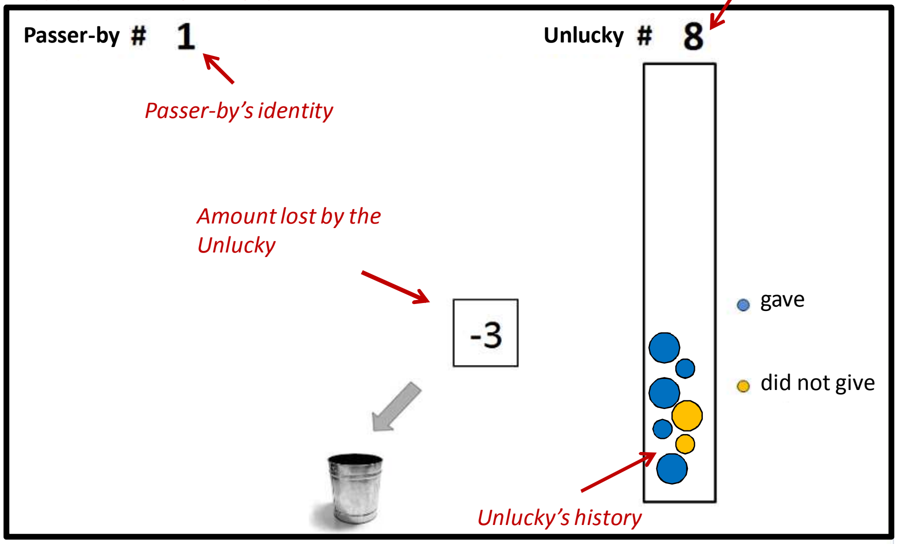

Example

Example where the Unlucky loses 5 CHF

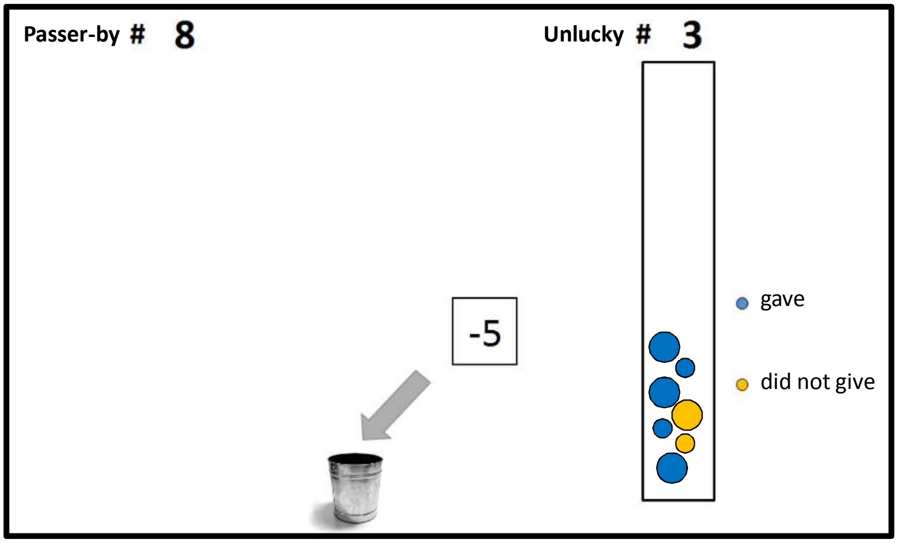

Example

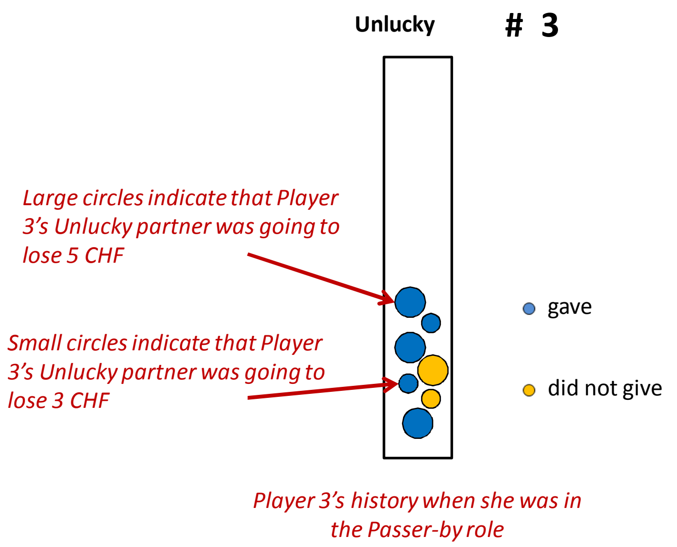

Example

Display when the Passer-by's decision is due

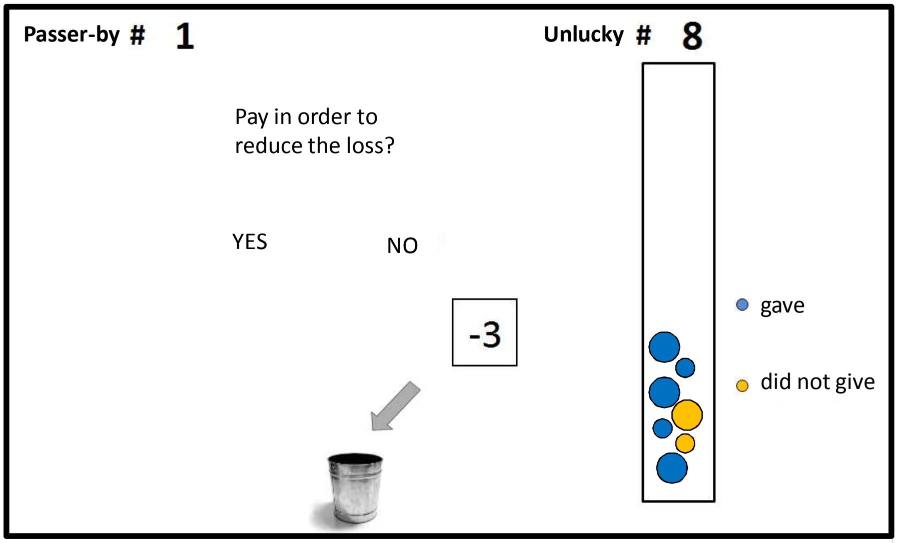

Example

Display when the Passer-by's has made her decision

Passer-by # 1 **Unlucky # 8**

Pay in order to reduce the loss?

YES NO

-1 -1

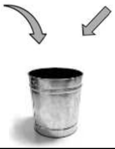

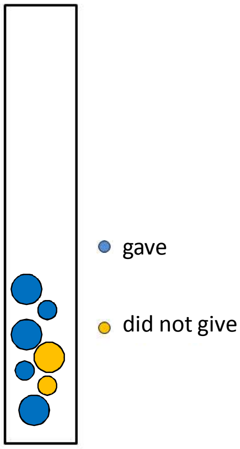

Example

Display when the Passer-by's has made her decision

Passer-by # 1 **Unlucky # 8**

Pay in order to reduce the loss?

YES NO

-1 -1

If the Passer-by says YES, she pays 1 CHF for the Unlucky to lose only 1 CHF

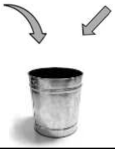

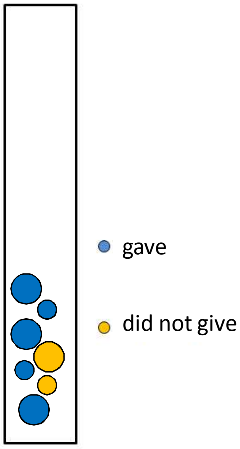

Example

Display when the Passer-by's has made her decision

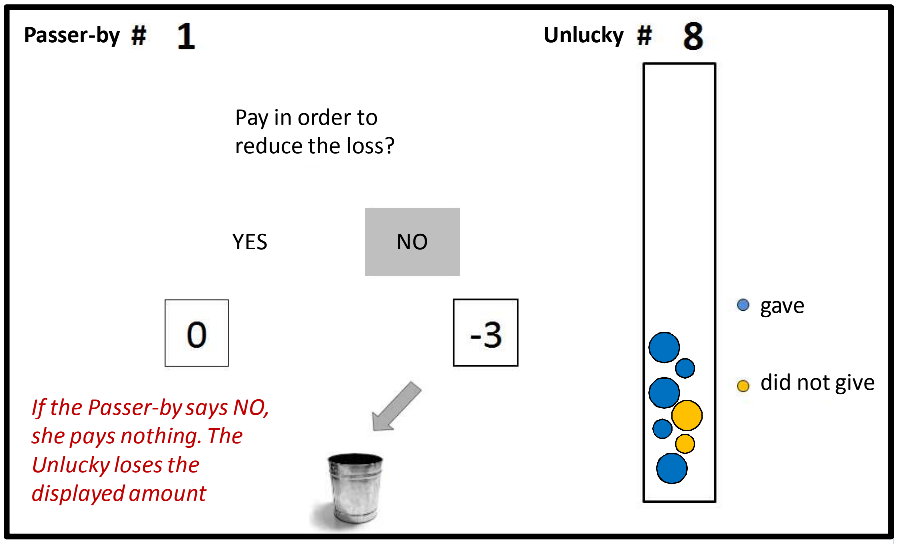

At the end of the game

- At the end of the game, you will be kindly asked to fill in the participation form and then place it in an envelope which will be sealed
- This envelope will only be opened by the accounting service of the Faculty of Biology in order to execute bank transfers
- You will learn about your final gains upon receipt on your bank account

26

Thanks for your attention !

Questions ?

27
